# Association between Single Nucleotide Polymorphisms and Weight Reduction in Behavioural Interventions—A Pooled Analysis

**DOI:** 10.3390/nu13030819

**Published:** 2021-03-02

**Authors:** Christina Holzapfel, Sabine Sag, Johanna Graf-Schindler, Marcus Fischer, Theresa Drabsch, Thomas Illig, Harald Grallert, Lynne Stecher, Christina Strack, Ian D. Caterson, Susan A. Jebb, Hans Hauner, Andrea Baessler

**Affiliations:** 1 Institute for Nutritional Medicine, School of Medicine, Technical University of Munich, 80992 Munich, Germany; j.graf-schindler@tum.de (J.G.-S.); theresa.drabsch@tum.de (T.D.); lynne.stecher@tum.de (L.S.); hans.hauner@tum.de (H.H.); 2 Clinic of Internal Medicine II, University Hospital of Regensburg, 93053 Regensburg, Germany; sabine.sag@ukr.de (S.S.); marcus.fischer@ukr.de (M.F.); christina.strack@ukr.de (C.S.); andrea.baessler@ukr.de (A.B.); 3 Hannover Unified Biobank, Hannover Medical School, 30625 Hannover, Germany; illig.thomas@mh-hannover.de; 4 Institute of Epidemiology, Helmholtz Zentrum München, German Research Center for Environmental Health, 85764 Neuherberg, Germany; harald.grallert@helmholtz-muenchen.de; 5 Boden Collaboration, Charles Perkins Centre, University of Sydney, Sydney, NSW 2006, Australia; ian.caterson@sydney.edu.au; 6 Nuffield Department of Primary Care Health Sciences, University of Oxford, Oxford OX2 6GG, UK; susan.jebb@phc.ox.ac.uk; 7 ZIEL Institute for Food and Health, Else Kröner-Fresenius-Center of Nutritional Medicine, School of Life Sciences, Technical University of Munich, 85354 Freising-Weihenstephan, Germany

**Keywords:** weight loss, weight loss program, SNP, genetic variant, genotype

## Abstract

Knowledge of the association between single nucleotide polymorphisms (SNPs) and weight loss is limited. The aim was to analyse whether selected obesity-associated SNPs within the fat mass and obesity-associated (*FTO*), transmembrane protein 18 (*TMEM18*), melanocortin-4 receptor (*MC4R*), SEC16 homolog B (*SEC16B*), and brain-derived neurotrophic factor (*BDNF*) gene are associated with anthropometric changes during behavioural intervention for weight loss. genetic and anthropometric data from 576 individuals with overweight and obesity from four lifestyle interventions were obtained. A genetic predisposition score (GPS) was calculated. Our results show that study participants had a mean age of 48.2 ± 12.6 years and a mean baseline body mass index of 33.9 ± 6.4 kg/m^2^. Mean weight reduction after 12 months was −7.7 ± 10.9 kg. After 12 months of intervention, the *MC4R* SNPs rs571312 and rs17782313 were significantly associated with a greater decrease in body weight and BMI (*p =* 0.012, *p* = 0.011, respectively). The investigated SNPs within the other four genetic loci showed no statistically significant association with changes in anthropometric parameters. The GPS showed no statistically significant association with weight reduction. In conclusion there was no consistent evidence for statistically significant associations of SNPs with anthropometric changes during a behavioural intervention. It seems that other factors play a more significant in weight management than the investigated SNPs.

## 1. Introduction

Obesity remains one of the largest challenges for global health. According to recent trends, global obesity prevalence will reach 18% in men and surpass 21% in women by 2025 [1]. The aetiology of obesity is complex and available management options are not always effective [2]. Medical treatment guidelines suggest a combination of dietary, physical, and behavioural changes of at least 6 to 12 months duration to achieve sustained weight loss [3]. Behavioural interventions are associated with modest mean weight losses after 12 months but there is considerable unexplained inter-individual heterogeneity in outcomes [4]. Heterogeneity may result from differences in adherence to the intervention but may also be attributable to the complex mechanisms of body weight regulation and genetic susceptibility. Better knowledge of the determinants of weight loss success is a prerequisite for the improvement of current treatment strategies in terms of personalised care.

A common hypothesis is that the inter-individual variance in weight loss is in some part attributable to genetic predisposition. To test this hypothesis obesity-related single nucleotide polymorphisms (SNPs) have been investigated for associations with weight loss during lifestyle interventions. The role of SNPs in obesity therapy has been shown in some studies for genes like peroxisome proliferator-activated receptor gamma (*PPARG*) [5,6], matrix metalloproteinase 2 (*MMP2*), Perilipin-1 (*PLIN1*), and metalloproteinase inhibitor 4 (*TIMP4*) [6], while others did not show evidence for an association between SNPs and weight loss [7,8,9]. A systematic review and meta-analysis on the effect of the fat mass and obesity-associated (*FTO*) gene on weight loss demonstrated that the response to weight loss intervention was not significantly different between *FTO* genotypes [10]. Overall, research focusing on the association between obesity-related genetic variants and weight loss in lifestyle interventions is limited, results are inconsistent and have not been replicated, while other limitations including small sample sizes, low statistical power, and small effect sizes also make results variable and inconsistent. 

To achieve more understanding of the association between genetic factors and weight loss, data from four weight loss intervention groups were pooled for the investigation of associations between selected SNPs and changes of anthropometric parameters (body weight, body mass index (BMI), waist circumference, fat mass) after 12 months of weight management.

## 2. Materials and Methods

### 2.1. Study Design

Data from two weight loss intervention studies (Weight Watchers (WW) Efficacy study and Regensburg Weight Loss study), each with two intervention arms, were analysed. The original study design of the WW study has been published elsewhere [11].

Briefly, the WW study was a parallel, multicentric, randomized controlled trial. Participants were recruited from primary care practices in Germany, Australia, and the United Kingdom. Eligible participants were adults (age ≥ 18 years) with a BMI between 27 and 35 kg/m^2^ and one additional risk factor for an obesity-related comorbidity. The first of the two treatment groups received advice from their primary care professional (standard care, SC) for 12 months about weight loss. The second group was allocated to a free membership of the commercially available WW program for 12 months, promoting a hypo-energetic, balanced diet using ProPoints for daily monitoring of food intake, physical activity, and community-based weekly group meetings.

The Regensburg Weight Loss study was conducted in Regensburg, Germany, in adults with overweight or obesity and a constant weight for the previous three months. This study comprises participants of the Optifast52 program (franchise holder Nestlé Inc., Switzerland), a 52-week lifestyle intervention consisting of four stages based on the four modules of dietetics, medical supervision, physical activity, and psychology [12]. The weight reduction therapy (Optifast) consisted of different diet stages with an initial 12-week formula diet and weekly group meetings for behavioural support. The comparator was a 12-month weight loss program (Other Weight loss, WL) under the supervision of a certified dietician, with weekly weight monitoring and increased physical activity implemented through a fitness plan. Both studies received ethics approval from the local ethical committees and all participants provided written informed consent. The WW study was registered in the ISRCTN registry under the number ISRCTN85485463.

### 2.2. Anthropometric Measurements and Blood Parameters

Anthropometric data were obtained for height (in meters) with a stadiometer, body weight (in kg), and fat mass (in kg) with a body composition analyser in the WW study (Tanita BC-418, Tanita Corporation Tokyo, Japan) or an impedance analyser in the Regensburg Weight Loss study (Nutriguard©-Impedance Analysis Apparatus, Data Input GmbH Darmstadt, Germany). Waist circumference (in cm) was measured midway between the lower rib margin and the upper border of the iliac crest. In the WW study systolic and diastolic blood pressure (mmHg) and heart rate (beats per minute) were measured using local standardized procedures whilst in the Regensburg Weight Loss study blood pressure was measured three times on both arms with at least 60 s in-between the measurements and heart rate was measured after a sitting period of 5 min.

Blood samples were collected after 12 h of fasting. Glucose was measured by the hexokinase (Modular DPE, Roche Diagnostics GmbH, Mannheim, Germany; Siemens Advia 2400, Siemens Australia, Bayswater, Australia) or glucose oxidase method in the WW study. Triglycerides and high- and low-density lipoprotein (HDL, LDL) cholesterol were measured by enzymatic colorimetric assays (Modular DPE, Roche Diagnostics GmbH, Mannheim, Germany; Vitros 5.1FS platform, Ortho Clinical Diagnostics Inc., Raritan, NJ, USA). Other clinical-chemical parameters were measured using commercially available methods (Siemens Advia Centaur, Siemens Advia 2400, Siemens Australia, Bayswater, Australia). In the WW study, all blood analyses were carried out by external certified labs (SYNLAB Holding Deutschland GmbH (Augsburg, Germany); Laverty Pathology (New South Wales, Australia); Northampton General Hospital (Northampton, UK)). In the Regensburg Weight Loss study, all blood parameters were measured in-house at the Institute of Clinical Chemistry and Laboratory Medicine of the University hospital of Regensburg using established methods.

### 2.3. Genotyping

Genotyping was performed for the SNPs showing the largest per allele change in BMI, detected in genome-wide meta-analysis [13], including the following SNPs: rs1558902 (*FTO* gene), rs939583 and rs7561317 (transmembrane protein 18 (*TMEM18*) gene), rs571312 and rs17782313 (melanocortin-4 receptor (*MC4R*) gene), rs543874 and rs10913469 (SEC16 homolog B (*SEC16B*) gene) and rs10767664 and rs16917237 (brain-derived neurotrophic factor (*BDNF*) gene) [13]. These five loci demonstrated clear evidence of independent association signals with obesity traits. The SNPs *FTO* rs1558902, *TMEM18* rs7561317, *MC4R* rs17782313, *SEC16B* rs10913469, and *BDNF* rs16917237 were included in the sequencing process of the WW study. In the Regensburg Weight Loss study, *FTO* rs1558902, *TMEM18* rs939583, *MC4R* rs571312, *SEC16B* rs543874, and *BDNF* rs10767664 were analysed. To reduce heterogeneity in the genetic background, only Caucasians were included.

In the WW study, samples were genotyped with the Mass ARRAY system using the iPLEX Gold Chemistry (Sequenom, San Diego, CA, USA) and analysed in a matrix-assisted laser desorption ionization time of flight mass spectrometer (MALDI TOF MS, Bruker Daltonik, Leipzig, Germany) [14]. In the Regensburg Weight Loss study, samples were genotyped using TaqMan Assays. The SNPs in the same genetic locus were all in high linkage disequilibrium (LD) to each other, defined as R^2^ above 0.8 [15].

For quality control, deviation from the Hardy-Weinberg equilibrium using the likelihood chi-squared test (1 degree of freedom) with a cut-off of *p* = 0.01 (Bonferroni correction for five tests) was calculated. All SNPs fulfilled the Hardy-Weinberg equilibrium.

The minor allele frequencies (MAF) were consistent with the 1000 Genomes Project Phase 3 in the CEU (Northern and Western European Ancestry) population. To check for the success of genotyping, the call rate was calculated for each SNP. In the WW study, all call rates were high (>94%). Since in the Regensburg Weight Loss study only data from participants with all SNPs successfully genotyped were available, no call rate was calculated.

### 2.4. Statistical Analysis 

Participants with full data on age, sex, and body weight after 12 months were considered for the present analysis (completer analysis). Persons with missing genotypes for one of the SNPs were excluded. Data from the four intervention groups were pooled and analysed together (“combined”), adding up to a total of 576 participants: 190 (WW), 192 (SC), 105 (Optifast) and 89 (Other WL). All investigated traits resembled a normal distribution. To test for associations between genotypes and parameters linear regression analyses were performed. The independent variable was either the occurrence of BMI-increasing risk alleles (1) or none (0) in the dominant model, the number of risk alleles (0, 1, 2) in the additive model, or the cumulative number of risk alleles (genetic predisposition score (GPS)). The GPS has been calculated for each participant by adding the risk alleles (0, 1, or 2) of all five variants according to Li et al. [16]. The lowest GPS was 0 (no risk allele over all five variants), and the highest GPS was 10 (meaning 10 risk alleles over all five variants). The baseline trait or the changes in anthropometric traits from baseline to 12 months were the outcome variables, adjusted for age, sex, and intervention group (and baseline body weight, respectively). For all regression analyses the beta coefficient (beta), as the regression coefficient, and the respective *p*-value (<0.05) for significance were calculated. The findings are reported with no correction for multiple testing as this was an exploratory study of the association between SNPs and weight loss from an intervention trial. The arithmetic mean (mean) and the standard deviation (SD) were calculated for the descriptive statistics. Statistical analysis was conducted using IBM SPSS Statistics 23 (Statistical Package for Social Science, IBM).

## 3. Results

### 3.1. Characteristics of Participants: Baseline and after 12 Months

The mean age was 48.2 ± 12.6 years (Table 1). Overall, the majority (77.6%) of participants were women. The overall mean body weight at baseline was 96.3 ± 24.2 kg. The baseline mean weight was lowest in the WW and SC groups (86.2 ± 11.6 kg and 86.7 ± 12.0 kg, respectively, *p* < 0.001) and highest in the Optifast group with 128.3 ± 31.3 kg. The BMI, fat mass, and waist circumference measurements showed similar distributions between the groups, with the lowest values in the WW and SC groups and highest in the Optifast group (comparison between intervention groups *p* < 0.001).

After 12 months, the overall mean weight change was −7.7 ± 10.9 kg. Weight loss was largest in the Optifast group (−22.2 ± 15.3 kg), followed by the WW group with −6.4 ± 6.2 kg. The SC and Other WL interventions led to similar mean weight changes (−3.1 ± 4.7 kg and −3.1 ± 7.3 kg, respectively). The overall minimum and maximum weight change were both in the Optifast group and ranged between −65.4 kg and +26.0 kg. Loss of BMI, waist circumference, and fat mass were also highest in the Optifast group and lowest in the SC and Other WL groups. All anthropometric differences between intervention groups were statistically significant *p* < 0.001. Blood parameters improved between baseline and the 12 months intervention. Systolic and diastolic blood pressure, as well as heart rate, decreased (Table 1).

### 3.2. Association between Genotypes and Baseline Anthropometric Traits

In the combined dataset with 576 participants in total, statistically significant associations between the investigated genetic variants and baseline anthropometric traits were seen for the *FTO* and *TMEM18* gene. The association of the risk allele A within the *FTO* gene (SNP rs1558902) with higher baseline body weight (beta = 2.90 kg; *p* = 0.049), higher baseline BMI (beta = 0.93 kg/m^2^; *p* = 0.017) and higher baseline fat mass (beta = 1.74 kg; *p* = 0.042) was seen in the unadjusted additive regression model. The *TMEM18* gene (SNPs rs939583 and rs7561317) was after adjustment for age and sex significantly associated with higher baseline body weight (beta = 4.75 kg; *p* = 0.002), higher baseline BMI (beta = 1.14 kg/m^2^; *p* = 0.015) and higher baseline fat mass (beta = 2.86 kg; *p* = 0.010) (adjusted additive model). The risk allele T within the *TMEM18* SNP rs939583 and the G risk allele in the *TMEM18* SNP rs7561317 were associated with higher anthropometric traits in the additive model. The further three investigated genes (*MC4R*, *SEC16B*, and *BDNF*) showed no significant association with baseline anthropometric traits.

### 3.3. Association between Genotypes and Changes in Anthropometric Traits

In the combined analysis over all four intervention groups, the *MC4R* gene (SNP rs571312 and rs17782313) was significantly associated with changes in body weight (beta = −1.39 kg; *p* = 0.012, additive model) and BMI (beta = −0.47 kg/m^2^; *p* = 0.011, additive model) after adjusting the regression model for age, sex, intervention group, and baseline weight. Thereby, the risk alleles (A in rs571312 and rs17782313) led to a larger loss of body weight and BMI after 12 months (Table 2). This association has been observed also in the dominant model. The other four investigated genes (*FTO, TMEM18, SEC16B, BDNF*) did not show any association with changes in anthropometric traits after 12 months (Table 2).

### 3.4. Associations between Genetic Predisposition Score (GPS) and Anthropometric Traits

The five investigated genes combined showed no significant association with baseline body weight (beta = 1.15 kg; *p* = 0.055) or waist circumference (beta = 0.80 kg/m; *p* = 0.052). The GPS also showed no significant association with baseline BMI (beta = 0.29 kg/m^2^; *p* = 0.114) or fat mass (beta = 0.67 kg; *p* = 0.110). 

**Figure 1:**
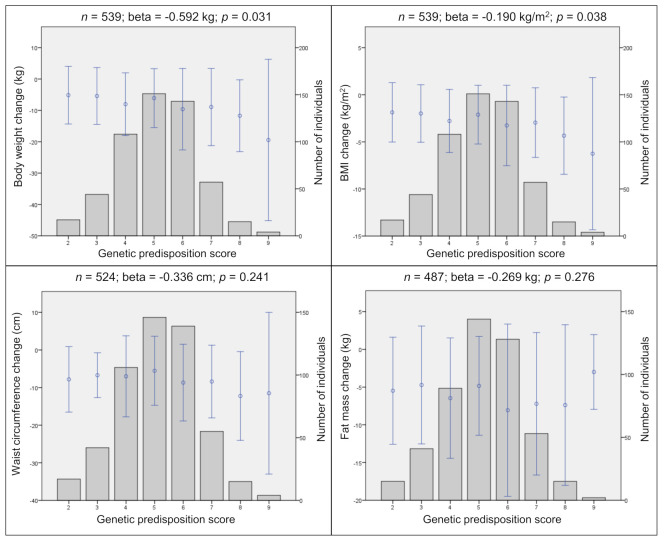
Genetic predisposition score (GPS) and changes in anthropometric traits. Genetic predisposition score (GPS) and cumulative effects of the risk alleles on changes in body weight, BMI, waist circumference, and fat mass in the combined data. Points represent the mean (± SD) of traits. Bars represent the distribution of GPS. Corresponding linear regression results are shown above each plot after 12 months. *n*, sample size; beta, regression coefficient; *p*, *p*-value; m, meter; cm, centimeter; kg, kilogram; SD, standard deviation. Linear regression adjusted for age, sex, intervention group, and baseline weight.

Statistically significant associations have been observed after 12 months. Thereby a higher GPS led to a higher body weight loss (beta = −0.59 kg; *p* = 0.031) and BMI loss (beta = −0.19 kg/m^2^, *p* = 0.038) (Figure 1).

However, these effects disappeared, when a subgroup analysis (weight loss ≥ 0 kg, *n* = 468) of individuals, excluding those with weight gain after 12 months, was performed. In this more homogenous group, the GPS showed no significant association with any of the changes in anthropometric traits after 12 months. All analyses were adjusted for age, sex, and intervention group.

## 4. Discussion

Associations between genetic variants and anthropometric traits at baseline and the changes after 12 months of behavioural intervention have been investigated. In a pooled analysis with 576 individuals, the *TMEM18* gene showed significant associations with anthropometric baseline data in the adjusted model. The *MC4R* gene (risk alleles within the SNPs rs571312 and rs17782313) was significantly associated with changes in body weight and BMI after 12 months. The calculation of the cumulative effect of risk alleles in five genetic variants within an individual GPS was not associated with anthropometric changes.

### 4.1. Genetic Analyses of Baseline Body Weight, BMI, Waist Circumference, and Fat Mass 

Each *TMEM18* gene risk allele (risk allele T of SNP rs939583 or risk allele G of rs7561317) led to a 4.8 kg higher body weight, a 1.1 kg/m^2^ higher BMI, and a 2.9 kg higher fat mass. However, the homozygous non-risk allele carrier group was very small (body weight and BMI, *n* = 14; fat mass, *n* = 13), which could have led to false-positive findings due to the small sample size. The association between *TMEM18* and obesity-related anthropometric parameters is in line with the literature [17,18].

The *FTO* gene (SNP rs1558902) showed significant associations with baseline body weight, BMI, and fat mass in the unadjusted model. After adjustment, the associations between *FTO* and baseline BMI remained borderline significant (*p* = 0.050). Thereby each risk allele A was significantly associated with a 0.668 kg/m^2^ higher baseline BMI.

The association of the *FTO* gene with BMI and missing associations of the other investigated genes (*MC4R, SEC16B, BDNF*) with any baseline anthropometric trait in the present work is contrary to the literature [10,13,19,20,21,22,23]. This can probably be explained by low statistical power and aggravated findings of genetic effects, due to a rather small sample size of the study population. Associations found in large observational studies with data from several hundred thousand individuals, may be challenging to replicate in the rather small sample sizes of interventional studies. 

### 4.2. Genetic Analyses of Changes in Body Weight, BMI, Waist Circumference, and Fat Mass

All interventions led to moderate weight loss, but with inter-individual differences. Ranges in weight loss were rather large (from 22.2 kg in the Optifast group to 3.1 kg in the SC and 3.1 kg in the Other WL group). This work found a significant association of the minor risk allele in the *MC4R* gene (A risk allele in rs571312 and C risk allele in rs17782313) with a 1.39 kg higher weight loss and 0.47 kg/m^2^ higher BMI loss. In another study in children, homozygous carriers of the minor C risk allele of the *MC4R* SNP rs17782313 lost significantly more weight than the other genotype groups after four weeks [24]. A study in the framework within the Diabetes Prevention Program investigated 20 SNPs within the *MC4R* gene for association with weight loss in 3,000 individuals [25]. One of the investigated SNPs within the *MC4R* gene (rs12970134) is in high LD with the SNPs rs17782313 and rs571312 from this analysis (*R*^2^ = 0.84 and 0.81, respectively). The SNP rs12970134 did not show any significant associations with changes in body weight after 6 or 24 months [25].

Variations in the *MC4R* locus were shown several times to be strongly associated with anthropometric traits [13,18,20]; nevertheless, the findings concerning *MC4R* genetic variants and changes in anthropometric traits remain controversial. Besides, effect sizes (shown as beta coefficient in this analysis) were rather high. Explanations might be the inclusion of individuals with weight gain on the one hand and individuals with extreme weight loss after 12 months on the other hand.

In this work, no significant association of the *FTO* SNP rs1558902 with changes in anthropometric traits was found. This is in line with literature investigating the association between the *FTO* SNP rs9939609 (in high LD with rs1558902, *R*^2^ = 0.92) and changes in anthropometric measurements after lifestyle intervention. Despite effects on appetite scores in a hypocaloric, high-protein diet intervention group [26] no significant association with weight loss between A risk allele carriers and non-risk allele carriers was shown [26,27], even though risk allele carriers showed a significantly lower body weight loss during the weight maintenance phase of the Optifast52 program [27]. Additional findings from eight other intervention studies with a total of approximately 10,000 participants were meta-analysed and showed that changes in anthropometric traits were not different between the *FTO* rs9939609 genotypes [10]. Evidence can be seen regarding the *FTO* rs9939609 risk allele and its effect on higher energy intake and reduced satiety in comparison to non-risk allele carriers [28]. However, a recent systematic review could not replicate an association between SNPs and macronutrient intake [29]. 

In the present study, no significant association between *BDNF*, *TMEM18*, and *SEC16B* and changes in anthropometric traits after 12 months could be shown. Existing literature on the same SNPs or SNPs in high LD to those analysed in this study showed no significant association with weight loss after 6 or 12 months in adults with overweight and obesity: rs6548238 of the *TMEM18* gene, rs6265 of the *BDNF* gene, rs10913469, and rs543874 of the *SEC16B* gene [6,30]. However, the analysis of weight loss rate (kg/year/allele) showed a significant association of the C risk allele in the *BDNF* SNP rs6265 with a higher weight loss rate [30].

Regarding genetic predisposition to obesity, the investigation of a cumulative effect of multiple SNPs seems to be a better approach than the analysis of single SNPs. In a study from China on more than 2800 persons, four out of 28 BMI-related SNPs showed significant associations with BMI, while the GPS was significantly associated with BMI and body fat [31]. The GPS analysis in this study was not associated with changes in any of the investigated traits (body weight, BMI, waist circumference, BMI). A similar analysis covering two SNPs within the *FTO* and *MC4R* gene (rs9936909 and rs17782313) analysed in the present study showed significant association with larger two months weight loss after adjustment for baseline weight [32]. The non-significance of the findings from the present work may be attributable to the small sample size, the heterogeneity of included studies, and the inclusion of only five SNPs in the GPS. This number of analysed SNPs is low compared to other GPS studies on baseline anthropometric traits [16,33], even though the study of Verhoef et al. [32] had a similar study design with as little as six obesity candidate genes from data of 150 adults. Characteristics of the study cohorts and differences in the statistical analysis may also contribute to inconsistent findings.

Even large-scale analyses might lack the statistical power to detect small genetic effects on weight loss or might show that there is no genetic effect on weight loss. In the combined analysis of the Diabetes Prevention Program and the Look Action for Health in Diabetes (AHEAD) study (almost 6,000 participants) in which associations between 93 independent SNPs and weight loss were analysed, the SNP rs1885988 within the melanogenesis associated transcription factor 3 (*MTIF3*) gene was significantly associated with weight loss across all four years of follow-up [34]. The Food4Me study [21] reported that A risk allele carriers of the *FTO* SNP rs9939609 lost significantly more body weight and waist circumference than non-risk allele carriers after six months [21]. Gardner et al. [35] reported, that the allocation of participants to a weight loss diet group according to their genotype showed no significant association and, therefore, the genotype did not help identify which diet is preferable for an individual to lose more weight [35].

### 4.3. Strengths and Limitations

The pooling of the data from four intervention groups for a combined analysis increased the statistical power by increasing the sample size. Besides, the five investigated genes were the genes with the highest per allele change in BMI found by investigation of over 120,000 individuals [13]. Furthermore, associations between genotypes and anthropometric traits were tested with two approaches of linear regression models. The additive model takes into consideration that within a SNP each additional risk allele has an additive effect on the outcome. Furthermore, the dominant model compares non-risk allele carriers to risk allele carriers of a specific SNP.

In all analyses, different adjustments were made to overcome possible confounding factors. Next to the common adjustments for age and sex in genetic association studies, also baseline body weight was also taken into consideration in analyses on changes of anthropometric traits to adjust for the heterogenous baseline BMI between intervention groups. To minimize the effect of the different interventions adjustments were made for the intervention group. Furthermore, in a sub-group analysis the association between genetic factors and baseline anthropometric traits as well as anthropometric changes has been investigated separately for each intervention group (data not shown). For all analyses, only Caucasians were included to reduce heterogeneity in the genetic background. As well, a GPS was calculated to investigate possible cumulative effects of genetic variants and to overcome the small effects [16].

Another strength is the inclusion of four obesity-associated measures in the analysis. Most studies focus on either BMI or body weight, but in this study body weight, BMI, waist circumference, and fat mass were investigated for possible associations with genetic variants. 

The investigated population represents a limited range of BMI, since only individuals with overweight and obesity were included in the intervention studies. Therefore, findings from baseline data are not transferable to the general population. Differences between the intervention groups, like sample size or design of the intervention, may influence the associations between genetic variants and changes in anthropometric traits. Results from gene-lifestyle (e.g., diet, physical activity) interactions, which were not calculated in the present study, might be of added value.

Only completers (individuals with data from baseline and 12 months available) were investigated. Therefore, no other measurement time points, between baseline and after 12 months, were taken into account, to correct for possible fluctuations between measurements. Furthermore, the completers analysis could lead to overestimation of weight loss after 12 months, since the most successful participants remained in the study. 

The basis for this analysis was a hypothesis-driven approach that genetic variants associated with obesity also show associations with weight loss. This is a very strong assumption, which might also be a reason for the non-significant findings in the present study. However, it could be possible that other genetic variants play a role in weight loss. Therefore, hypothesis-free GWAS focusing on the identification of genetic variants and weight loss would be of added value. Furthermore, the amount of weight loss is dependent on many factors (e.g., compliance, metabolism, physical activity) which might have a higher impact on successful weight management than genetic factors.

## 5. Conclusions

The findings from the present study provide no consistent statistically significant evidence for an association of the five investigated obesity-associated genes (*FTO, TMEM18, MC4R, SEC16B, and BDNF*) with baseline anthropometric traits or their changes after 12 months of behavioural intervention. The success in weight reduction might be more dependent on the intervention than on the genetic background studied here. To better elucidate potential influences of genetics on weight loss, larger intervention trials are needed. 

## Figures and Tables

**Table 1:** Characteristics of participants: baseline and after 12 months.

	Weight Watchers			Standard Care			Optifast			Other Weight Loss			All Combined		
	*n* = 190 88.4% Women			*n* = 192 83.9% Women			*n* = 105 51.4% Women			*n* = 89 71.9% Women			*N* = 576 77.6% Women		
	*n*	Mean	SD	*n*	Mean	SD	*n*	Mean	SD	*n*	Mean	SD	*n*	Mean	SD
**Baseline**															
Age (years)	190	48.73	12.92	192	51.25	11.99	105	45.09	12.51	89	44.42	11.51	576	48.24	12.59
**Anthropometry**															
Height (m)	190	1.65	0.08	192	1.66	0.09	105	1.71	0.10	89	1.70	0.08	576	1.67	0.09
Weight (kg)	190	86.17	11.60	192	86.74	12.02	105	128.26	31.34	89	101.01	19.60	576	96.32	24.15
BMI (kg/m^2^)	190	31.23	2.58	192	31.25	2.64	105	43.04	7.89	89	34.79	5.66	576	33.94	6.38
Fat mass (kg)	175	32.94	6.61	172	32.80	7.62	103	54.68	16.38	85	41.84	13.35	535	38.44	13.59
WC (cm)	188	98.84	8.50	189	100.16	9.94	103	126.50	17.86	88	107.52	15.85	568	105.64	16.04
**Clinical parameters**															
Systolic BP (mmHg)	190	125.66	16.76	192	125.48	15.03	103	142.72	16.84	88	134.35	13.92	573	130	17.13
Diastolic BP (mmHg)	190	78.12	9.89	192	79.50	8.63	103	88.53	13.07	88	85.9	9.69	573	81.86	10.90
Heart rate (bpm)	186	70.48	9.50	183	70.97	10.79	104	75.93	13.71	89	69.94	13.04	562	71.56	11.54
Glucose (mg/dl)	190	90.84	14.35	192	92.25	17.63	105	109.47	36.21	89	90.97	12.64	576	94.72	21.94
LDL (mg/dl)	182	125.04	35.41	188	125.68	33.60	105	123.90	32.80	89	122.16	33.23	564	124.58	33.92
HDL (mg/dl)	182	55.84	14.74	188	56.54	13.40	105	47.71	13.38	89	50.94	16.08	564	53.79	14.68
Triglycerides (mg/dl)	190	122.89	62.00	192	131.25	74.59	105	158.20	86.16	89	120.97	58.8	576	131.83	71.80
**Changes after 12 months**															
**Anthropometry**															
Δ Weight (kg)	190	–6.37	6.18	192	−3.14	4.74	105	−22.15	15.28	89	−3.12	7.28	576	−7.67	10.92
Δ BMI (kg/m^2^)	190	−2.29	2.19	192	−1.13	1.69	105	−7.37	4.76	89	−1.06	2.48	576	−2.64	3.58
Δ Fat mass (kg)	174	−5.03	5.26	166	−2.37	4.08	99	−16.64	12.37	80	−3.28	6.02	519	−6.13	8.74
Δ WC (cm)	181	−6.47	7.16	187	−4.22	6.31	103	−16.5	12.80	88	−4.27	9.61	559	−7.22	9.73
**Clinical parameters**															
Δ Systolic BP (mmHg)	187	−2.85	13.72	192	−1.95	13.94	102	−11.21	15.38	85	−0.42	12.98	566	−3.69	14.43
Δ Diastolic BP (mmHg)	187	−1.97	9.02	192	−1.55	9.32	102	−6.89	10.92	85	−1.38	9.58	566	−2.62	9.76
Δ Heart rate (bpm)	175	−1.12	10.42	174	0.40	9.90	104	−10.82	14.72	86	−1.75	12.35	539	−2.60	12.21
Δ Glucose (mg/dL)	183	−1.75	9.33	184	−0.37	12.81	105	−14.07	29.62	89	0.99	9.85	561	−3.17	16.98
Δ LDL (mg/dL)	175	−0.99	23.97	181	5.01	23.91	103	−5.53	35.53	89	−1.51	23.90	548	0.05	26.70
Δ HDL (mg/dL)	176	4.71	9.44	181	2.76	8.43	103	6.03	8.86	89	2.76	9.43	549	4.00	9.08
Δ Triglycerides (mg/dL)	182	−7.57	47.81	184	−6.09	60.59	105	−40.32	81.16	89	11.28	62.56	560	−10.23	63.52

*n/N*, sample size; mean, arithmetic mean; SD, standard deviation; Min, minimum value; Max, maximum value; BMI, Body Mass Index; WC, waist circumference; BP, blood pressure; LDL, low-density lipoprotein; HDL, high-density lipoprotein; m, meter; kg, kilogram; cm, centimeter; mmHg, millimeters of mercury; bpm, beats per minute; mg, milligram; dL, deciliter; Δ, delta.

**Table 2:** Associations between genotypes and changes in anthropometric traits.

Changes in Anthropometric Traits	Genotypes									Additive Model		Dominant Model	
		0			1			2					
	*n*	Mean	SD	*n*	Mean	SD	*n*	Mean	SD	Beta	*p*-Value	Beta	*p*-Value
***FTO*—rs1558902**													
Δ Weight (kg)	158	−7.32	11.25	287	−7.31	9.82	117	−9.68	13.08	−0.376	0.468	−0.380	0.635
Δ BMI (kg/m^2^)	158	−2.50	3.64	287	−2.54	3.25	117	−3.30	4.28	−0.160	0.357	−0.185	0.490
Δ WC (cm)	153	−6.84	9.49	279	−7.11	9.48	113	−8.54	10.88	−0.390	0.475	−0.594	0.480
Δ Fat mass (kg)	140	−5.59	8.34	260	−6.20	7.98	107	−7.21	10.93	−0.339	0.464	−0.665	0.355
***TMEM18—*rs939583 and rs7561317**													
Δ Weight (kg)	14	−4.36	9.03	137	−7.41	8.83	404	-8.07	11.76	-0.440	0.541	−1.508	0.516
Δ BMI (kg/m^2^)	14	−1.54	2.92	137	−2.62	3.09	404	−2.75	3.81	−0.113	0.640	−0.494	0.525
Δ WC (cm)	14	−6.03	8.19	135	−7.38	9.49	389	−7.41	10.04	0.000	1.000	−0.164	0.946
Δ Fat mass (kg)	13	−4.7	7.61	122	−6.10	7.50	365	−6.37	9.28	0.266	0.679	−0.097	0.962
***MC4R—*rs571312 and rs17782313**													
Δ Weight (kg)	300	−6.64	10.05	224	−8.46	11.26	48	−10.58	13.95	−1.385	**0.012**	−1.612	**0.024**
Δ BMI (kg/m^2^)	300	−2.28	3.31	224	−2.94	3.70	48	−3.55	4.43	−0.472	**0.011**	−0.564	**0.019**
Δ WC (cm)	292	−6.63	9.14	216	−7.68	10.03	47	−8.84	11.78	−0.669	0.250	−0.752	0.318
Δ Fat mass (kg)	274	−5.63	8.00	201	−6.60	9.37	40	−7.55	10.39	−0.536	0.286	−0.584	0.361
***SEC16B—*rs543874 and rs10913469**													
Δ Weight (kg)	375	−7.38	10.74	174	−8.35	11.30	23	−7.55	11.89	−0.280	0.658	−0.534	0.477
Δ BMI (kg/m^2^)	375	−2.57	3.54	174	−2.85	3.74	23	−2.44	3.40	−0.048	0.820	−0.138	0.582
Δ WC (cm)	361	−7.02	9.82	172	−7.59	9.69	22	−7.64	9.12	−0.202	0.761	−0.317	0.687
Δ Fat mass (kg)	332	−5.94	8.66	162	−6.71	9.02	21	−5.30	8.50	−0.396	0.482	−0.687	0.302
***BDNF—*rs10767664 and rs16917237**													
Δ Weight (kg)	27	−5.76	10.30	200	−8.22	10.43	335	−7.56	11.43	0.065	0.916	−1.775	0.295
Δ BMI (kg/m^2^)	27	−2.08	3.29	200	−2.83	3.47	335	−2.59	3.72	0.052	0.801	−0.450	0.428
Δ WC (cm)	26	−6.48	9.17	193	−7.27	9.23	328	−7.23	10.11	0.033	0.959	0.133	0.940
Δ Fat mass (kg)	25	−5.16	9.04	175	−6.62	9.02	308	−5.96	8.68	0.077	0.888	−0.942	0.528

Beta, regression coefficient; Mean, arithmetic mean; *n*, sample size; SD, standard deviation; WC, waist circumference; BMI, Body Mass Index; kg, kilogram; m, meter; cm, centimeter; *FTO*, fat mass and obesity associated; *TMEM18*, transmembrane protein 18; *MC4R*, melanocortin-4 receptor; *SEC16B*, SEC16 homolog B; *BDNF*, brain-derived neurotrophic factor; Δ, delta. Additive model: Number of risk alleles (0, 1, 2), Dominant model: Occurrence of BMI-increasing risk alleles (1) or none (0). Both models were adjusted for age, sex, baseline weight, and intervention group. Bold *p*-values are considered statistically significant (*p* < 0.05).

## Data Availability

The data presented in this study are available on request from the corresponding author. The data can be shared with cooperation partners upon request.
